# Unveiling Community Vulnerability to COVID-19 Incidence: A Population-Based Spatial Analysis in Clark County, Nevada

**DOI:** 10.3390/ijerph22030326

**Published:** 2025-02-22

**Authors:** Lung-Chang Chien, L.-W. Antony Chen, Chad L. Cross, Edom Gelaw, Cheryl Collins, Lei Zhang, Anil T. Mangla, Cassius Lockett

**Affiliations:** 1 Department of Epidemiology and Biostatistics, University of Nevada, Las Vegas, NV 89154, USA; lung-chang.chien@unlv.edu (L.-C.C.); chad.cross@unlv.edu (C.L.C.);; 2 Department of Environmental and Occupational Health, University of Nevada, Las Vegas, NV 89154, USA; 3 Desert Research Institute, Las Vegas, NV 89119, USA; 4 Southern Nevada Health District, Las Vegas, NV 89107, USA

**Keywords:** COVID-19 disparity, community vulnerability index, lagged weighted quantile sum

## Abstract

Community vulnerability is influenced by various determinants beyond socioeconomic status and plays a crucial role in COVID-19 disparities. This study aimed to develop and evaluate a novel community vulnerability index (CVI) related to temporal variations in COVID-19 incidence to provide insights into spatial disparities and inform targeted public health interventions in Clark County, Nevada. Utilizing data from the American Community Survey and other sources, 23 community measures were identified at the census tract level. The CVI was constructed using a lagged weighted quantile sum (LWQS) regression linking these measures to the monthly COVID-19 incidence from March 2020 to November 2021. The Besag–York–Mollié model subsequently evaluated the spatial association between the CVI and COVID-19 incidence, controlling for temporal and spatial autocorrelations. This study identified minority status, housing inadequacy, and inactive commuting as primary contributors to the CVI that consistently influenced COVID-19 vulnerability over time. The CVI demonstrated significant spatial disparities, with higher values found in northern Clark County and the northeastern Las Vegas metropolitan area. Spatial analyses revealed varying associations between COVID-19 incidence and the CVI across census tracts, with significant associations clustered in the northern and eastern regions of the Las Vegas metropolitan area. These findings advance our understanding of the complex interplay between community conditions and COVID-19. The CVI framework may be applied to other COVID-19 outcomes such as testing, vaccination, and hospitalization, offering a valuable tool for assessing and addressing community vulnerability.

## 1. Introduction

The COVID-19 pandemic has remained an ongoing public health concern in the United States (U.S.) since the U.S. Centers for Disease Control and Prevention (CDC) reported the first confirmed case on 20 January 2020 [1]. Since then, the virus has continued to evolve, resulting in multiple waves of outbreaks across the country [2]. Research has demonstrated significant COVID-19 disparities driven by social conditions. In the U.S., the COVID-19 mortality rate is five times higher among adults with lower socioeconomic status compared to those with higher socioeconomic status [3]. Socioeconomic factors, such as the inability to adhere to social distancing, work from home, access sanitary supplies, and reduce reliance on public transportation, have been associated with increased COVID-19 incidence rates [4,5,6,7]. However, the dynamic relationship between social vulnerability and COVID-19 disparity over time warrants further investigation.

Social vulnerability refers to the susceptibility of certain individuals or groups to experience disproportionately negative impacts when faced with hazards or adverse events due to their social conditions [8]. It is a multifaceted construct influenced by various social determinants beyond socioeconomic status. In 2014, the CDC created a Social Vulnerability Index (SVI) that is calculated at both the census tract and county levels and has since updated it bi-annually [9]. The SVI is the sum of the percentile ranks of 16 social condition variables and, therefore, weights these variables equally [10]. Several studies have explored the impacts of the SVI on human health (e.g., cardiovascular disease and unintentional fatal injuries) [11,12] or medical treatment (e.g., cholecystectomy) [13]. Increases in SVI scores were also shown to be associated with increases in COVID-19 incidence and mortality rates [14].

As the importance of each social condition in the SVI likely varies with health outcomes, efforts have been made to refine the SVI to better target specific public health outcomes. These include considering additional community indicators beyond the 16 original variables, such as the female percentage [15], divorce rate [16], resident population density [17], minimum level of education [18], and unemployment rate [19], and/or using more sophisticated statistical methods, such as factor analysis [18,20], principal component analysis [21], the analytic hierarchy process [22,23], multi-criteria analysis [24], and machine learning [25], to derive the weights of the community variables considered in the model. Refinement of the SVI should also recognize that the weights can change over time, even with static community variables, due to external factors like policy interventions, behavioral patterns, or the dynamic nature of an epidemic.

This study aimed to construct a novel community vulnerability index (CVI) for COVID-19 on a monthly basis while investigating its primary contributing variables and its spatial association with COVID-19 incidence. The study area included the entirety of Clark County, Nevada. The CVI not only provided insights into the spatial disparity of COVID-19 incidence within Clark County but also identified communities where local factors could play an important role during the pandemic, thus informing health agencies and allowing further targeted research and interventions.

## 2. Materials and Methods

### 2.1. Study Area

**Figure 1:**
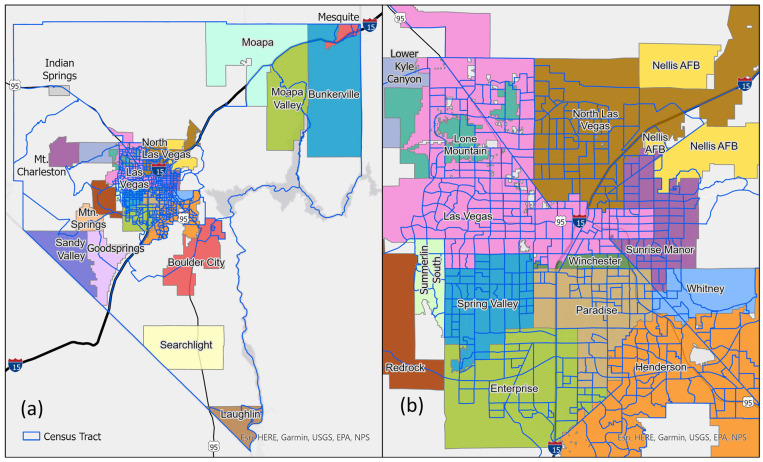
Maps of (**a**) Clark County and (**b**) the Las Vegas metropolitan area with the census tracts marked by blue lines.

Situated in southern Nevada, Clark County is the 13th largest county in the United States. It contains the world-famous Las Vegas Strip and has a population of over 2.2 million, according to the 2020 census. Additionally, it draws in more than 40 million visitors in a typical year. Clark County consists of 535 census tracts, most of which are located in the Las Vegas metropolitan area, which includes three incorporated cities, Las Vegas, North Las Vegas, and Henderson, as well as several townships and contains most of the population of the county (Figure 1).

### 2.2. Data Sources

Community variables were obtained from the following reputable public data sources: the American Community Survey (a 5-year estimate from 2016 to 2020) [26], the Smart Location Database [27], and Comprehensive Housing Affordability Strategy data [28]. COVID-19 incidence data were obtained from the Southern Nevada Health District, with the first COVID-19 case in Clark County reported on 5 March 2020. All COVID-19 cases were de-identified, geocoded, and aggregated by census tract and by month from March 2020 to November 2021. Reinfection cases were excluded from this study.

### 2.3. Variable Definitions

This study used 23 community measures to construct the CVI (Table 1). Fifteen of them overlapped with those identified in the CDC’s SVI [10]. In addition, the percentage of the household cost burden in the SVI was not used, as it was substituted by two measures specific to the percentages of low-income owner and renter households with housing costs exceeding 50% of their incomes. The other six variables were inactive commuting (the percentage of workers aged 16 and older who did not commute by transit, walking, or cycling), park deprivation (the percentage of the population residing more than half a mile from a park, beach, or open space larger than 1 acre), retail density (the employment density of retail, entertainment, service, and education jobs per acre on unprotected land), inadequate housing (the percentage of households without kitchen facilities and plumbing), segregation measured by the index of dissimilarity [29], and population density (the number of residents divided by the area in square miles). These community measures were inherently expressed in different units (e.g., percentages, jobs per acre, index scores, and persons per square mile) and exhibited varying distributions, which could have affected the robustness of the CVI. To address this challenge, all community measures were transformed into percentile ranks across the 535 census tracts, standardizing their values to a range from 0 to 1 (median = 0.5). This transformation was consistent with the methodology used for the CDC’s SVI [10].

### 2.4. Statistical Analyses

We first utilized a lagged weighted quantile sum (LWQS) regression to generate the CVI from the 23 chosen variables by linking them to the monthly COVID-19 incidence [30]. Like its precedent, the weighted quantile sum regression [31], the LWQS regression used the same bootstrapping technique to estimate the weight of each community variable with the constraint that the sum of all weights was equal to 1. The CVI was then defined as a weighted linear function of the quintiles of the 23 community variables. As the COVID-19 incidence (i.e., the outcome variable in the LWQS regression) changed by month, the estimated weights varied by month, leading to a time-varying CVI. The community variables were considered primary contributors to the CVI if their weights were larger than 1/23 = 0.0434, the fair share of their weights.

We applied the Besag–York–Mollié (BYM) model to quantify spatially varying associations between the CVI and COVID-19 incidence by evaluating an interaction term between the CVI and a spatial function derived from Markov random fields [32]. To control for temporal and spatial autocorrelations, our BYM model also included a linear term for the calendar time variable ranging from 1 (i.e., March 2020) to 21 (i.e., November 2021), a separate spatial function derived from Markov random fields, and a random intercept for the census tract effect. An offset term derived from the logarithm of the census tract population was also appended behind the model. All unknown parameters were estimated by the integrated nested Laplace approximation, an alternative approach to Bayesian inference that aimed to generate fast and accurate approximations of the posterior distribution [33]. The model’s reliability was confirmed through validation using conditional predictive ordinates [34].

The estimated spatial function was further transformed into relative risks (RRs) through an exponential function to epidemiologically explain the association between the CVI and COVID-19 incidence by census tract. The census tracts exhibited significant positive or negative associations between the CVI and COVID-19 incidence if the corresponding RRs’ 95% confidence intervals (CIs) were exclusively above or below 1, respectively.

Data were cleaned and managed using SAS v9.4 (SAS Institute Inc., Cary, NC, USA). Statistical analyses and mapping were conducted using RStudio 2022.07.1+554 (RStudio Inc., Boston, MA, USA). The significance level was set to 5%.

## 3. Results

The summary statistics of the 23 community variables are presented in Table 1. The coefficients of variation for these variables spanned from 0.07 (the percentages of inactive commuting and park deprivation) to 8.70 (population density), highlighting substantial diversity in their distributions across Clark County. While narrower distributions suggested greater uniformity across census tracts, the transformation to percentile ranks still allowed for meaningful rankings of the census tracts. Notably, after transforming all variables into percentile ranks, they were standardized to the same range (0–1) with uniform distributions, facilitating the calculation of the CVI through the LWQS regression.

**Figure 2:**
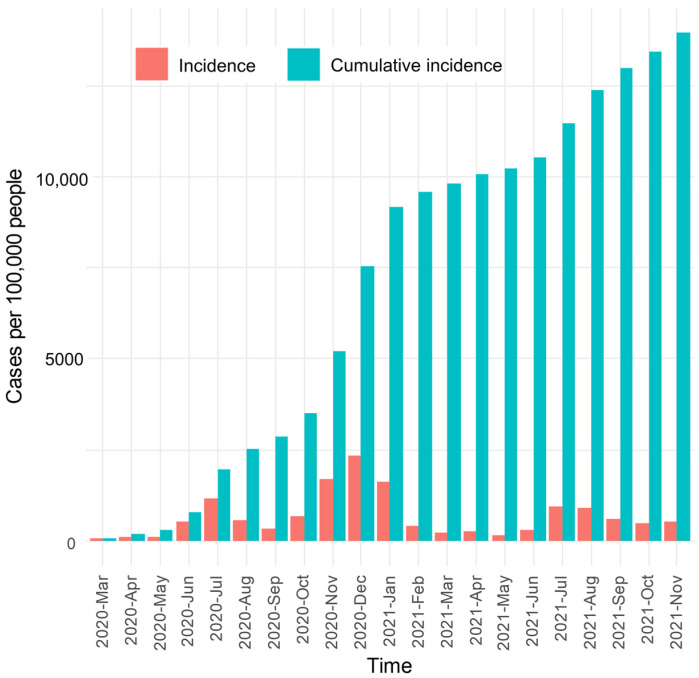
Temporal variation in COVID-19 incidence and cumulative incidence rates.

Figure 2 shows three peaks in the monthly COVID-19 incidence. The first peak occurred in July 2020, with 1153.05 cases per 100,000 people. The second peak occurred in December 2020, with 2321.76 cases per 100,000 people. The third peak occurred in July 2021, with 931.64 cases per 100,000 people. The first peak followed the lifting of a statewide lockdown, while the second peak was an anticipated winter surge right before the vaccination campaign starting in December 2021. The emergence of the Delta variant was consistent with the third peak in the summer of 2021. Eventually, the cumulative COVID-19 incidence rate reached 13,960.28 cases per 100,000 people in Clark County by November of 2021.

Table 2 summarizes the weight of each community variable across the 21 months. The top three average weights were observed for minority status (mean = 0.18; SD = 0.10), housing inadequacy (mean = 0.16; SD = 0.06), and inactive commuting (mean = 0.15; SD = 0.10). Minority status was the only variable also adopted in the CDC’s SVI, which was a primary contributor to the CVI in 18 of 21 months (85.71%). Inadequate housing and inactive commuting were primary contributors in all 21 months. Other variables that were primary contributors in at least 12 months included being 17 or younger (N = 17; 80.95%), park deprivation (N = 15; 71.43%), and not having a high school diploma (N = 14; 66.67%). On the contrary, seven community variables (below 150% poverty, civilian unemployment, households with more people than rooms, households with no vehicle available, retail density, and low-income homeowners with severe housing cost burdens) barely contributed to the CVI in all months.

**Figure 3:**
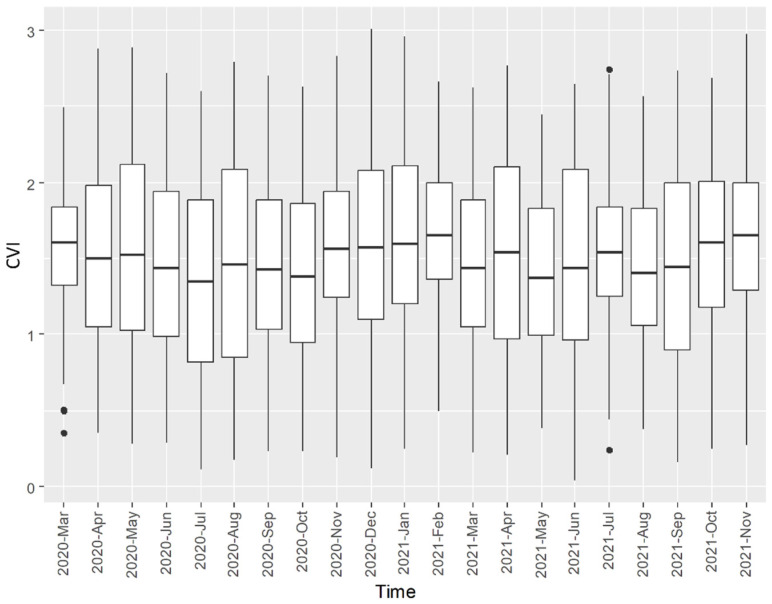
A summary of the CVI related to COVID-19 incidence in Clark County, Nevada, from March 2020 to November 2021. Outliers (dots) were displayed if their CVIs fell outside 1.5 times the interquartile range.

**Figure 4:**
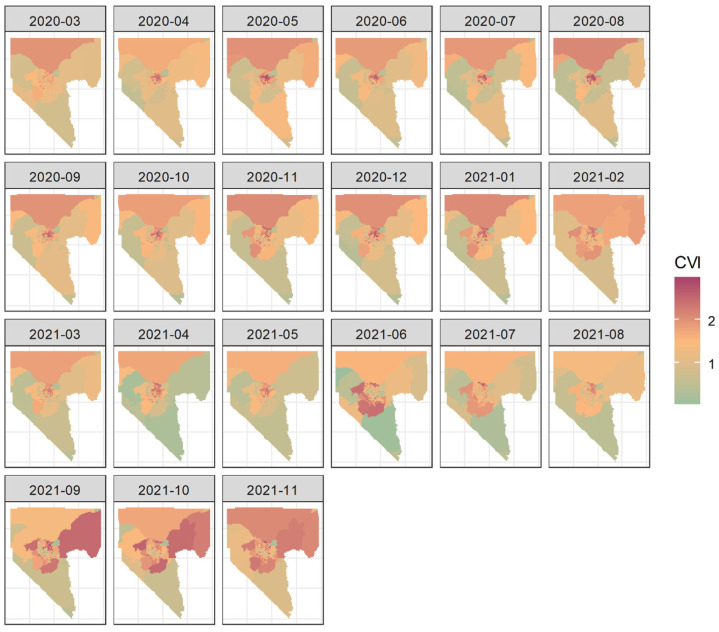
The geospatial distribution of the CVI related to COVID-19 incidence.

Figure 3 summarizes the CVI by month. The average CVI was between 1.37 (SD = 0.64; July 2020) and 1.67 (SD = 0.44; February 2021), reflecting the variations in the CVI’s spatial distribution. The geospatial distribution of the CVI from March 2020 to November 2021 is shown in Figure 4, indicating that higher CVI values were more likely to appear in census tracts located in northern Clark County and the northeastern Las Vegas metropolitan area. In the second half of 2021, higher-CVI areas expanded to the outskirts of the Las Vegas metropolitan area and northeastern Clark County.

**Figure 5:**
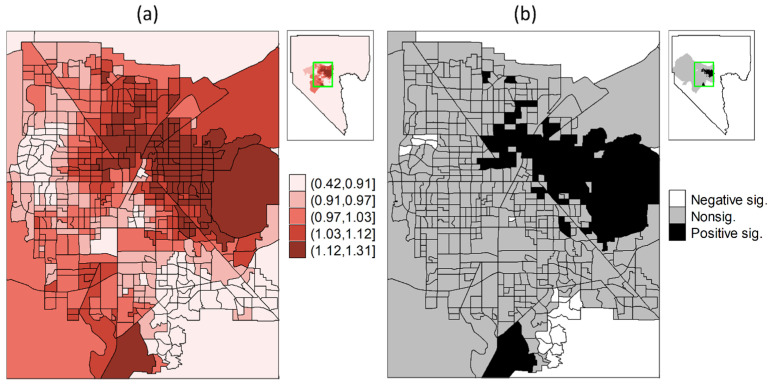
The spatial distributions of (**a**) the relative risks of COVID-19 incidence and (**b**) the corresponding significance derived from the estimated spatial function interacting with the CVI through the BYM model.

Regarding the association between the CVI and COVID-19 incidence by census tract, the BYM-estimated RRs were between 0.42 (95% CI = 0.28, 0.64) and 1.32 (95% CI = 1.16, 1.49). Higher RRs were observed in the northern and eastern parts of the Las Vegas metropolitan area, while rural census tracts generally had lower RRs (Figure 5a). Among the 535 census tracts, a total of 119 census tracts had significant RRs, including 84 census tracts (15.70%) with significant RRs > 1, all found in the northern and eastern Las Vegas metropolitan area (Figure 5b). In total, 35 census tracts (6.54%) had significant RRs < 1, mostly located in the southern and western outskirts surrounding the Las Vegas metropolitan area.

## 4. Discussion

This study developed a novel index to quantify community vulnerability specifically related to COVID-19 incidence in Clark County, Nevada, with the added feature of variability across different months. This research identified community measures, such as the percentages of inadequate housing and inactive commuting, that consistently played significant roles in influencing the CVI, though their weights changed over time as the pandemic progressed. The higher CVI values observed in northern Clark County and the northeastern Las Vegas metropolitan area align with previous environmental justice assessments identifying these areas as disadvantaged and underserved [35]. This disadvantage has primarily been attributed to the prevalence of poverty, housing and infrastructure deficiencies, and a high proportion of minority populations. We further assessed the geospatial disparities of the derived CVI by examining its association with COVID-19 incidence using the BYM model. The results show that the CVI explains COVID-19 incidence better in some communities than in others, as evidenced by stronger associations in certain census tracts. Weaker or insignificant associations in other census tracts imply additional local factors that warrant further investigation. These findings underscore the dynamic and multifaceted nature of social vulnerability to viral transmission.

Various studies have emphasized the importance of accurately assessing vulnerability to understand and address disparities under the impact of the COVID-19 pandemic. Several vulnerability indices have been developed, though most of them do not include COVID-19 information, so they, just like the CDC’s SVI, are not specific to COVID-19. For instance, a vulnerability index combined 41 equally weighted socioeconomic, environmental, healthcare, and epidemiological variables from eight themes in 21 counties in New Jersey and showed a better capability than the CDC’s SVI for predicting COVID-19 incidence and mortality [36]. Another vulnerability index was built by a weighted function to combine 17 epidemiological and healthcare system variables in 35.57% of U.S. counties, with their weights estimated by subcomponents from a principal component analysis. It revealed significant associations with COVID-19 transmissibility [37]. Such indices may be refined by better relating them with COVID-19 outcomes. In addition, these studies assumed static weights for contributing variables and uniform impacts for the vulnerability indices on COVID-19 outcomes, which have not been tested adequately [38].

The novelty of this research was that it demonstrated the feasibility of customizing a time-varying vulnerability index through a theoretically supportive modeling tool. Similar indices have not been built at the census tract level to reveal fine spatial variations within a county. Future work could be expected if data, not limited to COVID-19, become available to assess CVIs specific to subpopulations (e.g., genders, races, and ages) and identify vulnerable areas. Such findings would inform local health agencies and allow further targeted public health interventions.

While this study identified primary contributors to the CVI, which may facilitate the design of intervention strategies, these findings should be carefully explained. The index weights estimated from the LWQS regression only reveal how much they contribute to the index. Therefore, the weights should not be interpreted as definitive associations between the community variables and COVID-19 incidence. The low weights assigned to “below 150% poverty” and “civilian unemployment”, for instance, do not necessarily indicate that these factors have no effect on COVID-19 incidence. Rather, they suggest that these factors contribute less to the CVI compared to variables such as inadequate housing and inactive commuting. Moreover, while the time-varying weights of the CVI variables likely reflect the effects of public health interventions (e.g., lockdowns, mask mandates, and vaccination programs) at different time points, it is challenging to provide statistical proof of these effects within the LWQS framework.

## 5. Conclusions

This research advanced our understanding of the complex interplay between community conditions and COVID-19. By developing a novel CVI related to COVID-19 incidence that considers monthly variations, this study identified determinants such as inadequate housing and inactive commuting as pervasive factors that contribute to COVID-19 vulnerability. Moreover, this study recognized regional disparities, highlighting that the COVID-19 incidence attributable to the CVI varied across the census tracts. These findings identified communities where further investigations into influencing factors beyond the CVI are warranted while emphasizing the need for tailored public health interventions in the fight against the COVID-19 pandemic. Our analytic strategy may be applied to study spatial surveillance data related to other COVID-19 measurements, such as vaccination or hospitalization.

## Figures and Tables

**Table 1:** Summary statistics of the selected community variables.

Variable	Mean	SD	CV	Min	IQR	Max
Below 150% poverty (%)	22.55	14.26	0.63	0.00	20.00	74.70
Civilian (age 16+) unemployment (%)	6.69	4.31	0.64	0.00	5.40	29.20
No high school diploma (age 25+) (%)	13.56	11.07	0.82	0.00	12.80	59.80
Uninsured (%)	11.23	7.46	0.66	0.00	10.20	50.10
Aged 65 and older (%)	17.10	12.01	0.70	0.00	11.10	100.00
Aged 17 and younger (%)	21.78	7.65	0.35	0.00	10.40	41.80
Disability (%)	13.11	5.80	0.44	0.00	7.00	38.40
Single-parent households with children under 18 (%)	6.96	5.15	0.74	0.00	6.60	31.50
Individuals speak English “less than well” (%)	5.38	6.22	1.16	0.00	6.50	36.20
Minority (%)	49.83	22.68	0.46	0.00	35.80	96.60
Housing in structures with 10 or more units (%)	13.04	17.94	1.38	0.00	19.20	99.00
Mobile homes (%)	5.77	12.74	2.21	0.00	3.40	76.20
Households with more people than rooms (%)	4.31	4.82	1.12	0.00	5.60	30.60
Households with no vehicle available (%)	7.31	9.09	1.24	0.00	8.10	55.80
Group quarters (%)	1.27	5.99	4.72	0.00	0.20	87.90
Inactive commuting (%)	95.11	6.52	0.07	31.48	5.43	100.00
Park deprivation (%)	97.32	6.35	0.07	34.09	2.73	100.00
Retail density (jobs/acre)	12.01	57.89	4.82	0.00	9.20	1075.72
Low-income homeowners with severe housing cost burdens (%)	5.88	5.28	0.90	0.00	7.19	34.78
Low-income renters with severe housing cost burdens (%)	16.52	12.41	0.75	0.00	19.44	60.42
Inadequate housing (%)	69.77	44.25	0.63	0.00	90.00	100.00
Segregation based on index of dissimilarity (point)	32.87	12.66	0.39	0.17	15.88	98.69
Population density (persons/mile^2^)	15.07	131.07	8.70	0.13	0.54	2182.76

Mean: simple average; SD: standard deviation; CV: coefficient of variation; Min: minimum; IQR: interquartile range; Max: maximum.

**Table 2:** A summary of the monthly weights estimated by the lagged weighted quantile sum regression from March 2020 to November 2021.

Variable	Mean	SD	IQR	Primary Contributor # (%) *
Below 150% poverty	0.00	0.00	0.00–0.00	0 (0.00)
Civilian (age 16+) unemployment	0.00	0.00	0.00–0.00	0 (0.00)
No high school diploma (age 25+)	0.06	0.05	0.03–0.09	14 (66.67)
Uninsured	0.01	0.02	0.00–0.02	4 (19.05)
Aged 65 and older	0.02	0.03	0.00–0.01	2 (9.52)
Aged 17 and younger	0.11	0.10	0.05–0.12	17 (80.95)
Disability	0.01	0.01	0.00–0.01	1 (4.76)
Single-parent households with children under 18	0.01	0.02	0.00–0.02	1 (4.76)
Individuals speak English “less than well”	0.06	0.06	0.00–0.10	10 (47.62)
Minority	0.18	0.10	0.13–0.24	18 (85.71)
Housing in structures with 10 or more units	0.01	0.02	0.00–0.01	1 (4.76)
Mobile homes	0.02	0.03	0.00–0.04	4 (19.05)
Households with more people than rooms	0.00	0.00	0.00–0.00	0 (0.00)
Households with no vehicle available	0.00	0.00	0.00–0.00	0 (0.00)
Group quarters	0.01	0.02	0.00–0.02	2 (9.52)
Inactive commuting	0.15	0.10	0.07–0.18	21 (100.00)
Park deprivation	0.11	0.10	0.04–0.16	15 (71.43)
Retail density	0.00	0.01	0.00–0.00	0 (0.00)
Low-income homeowners with severe housing cost burdens	0.01	0.01	0.00–0.02	0 (0.00)
Low-income renters with severe housing cost burdens	0.01	0.02	0.00–0.01	2 (9.52)
Inadequate housing	0.16	0.06	0.11–0.21	21 (100.00)
Segregation	0.02	0.02	0.00–0.04	4 (19.05)
Population density	0.00	0.00	0.00–0.00	0 (0.00)

* The number and percentage of months with weights > 0.0434 during the study period.

## Data Availability

The codes used in this study are available upon request from the corresponding author. The census-tract-aggregated COVID-19 incidence data are not publicly available due to the ethical and legal restrictions of the agency.
